# Single nucleotide seed modification restores *in vivo* tolerability of a toxic artificial miRNA sequence in the mouse brain

**DOI:** 10.1093/nar/gku979

**Published:** 2014-10-20

**Authors:** Alex Mas Monteys, Ryan M. Spengler, Brett D. Dufour, Matt S. Wilson, Clayton K. Oakley, Matt J. Sowada, Jodi L. McBride, Beverly L. Davidson

**Affiliations:** 1 The Center for Cell and Molecular Medicine, The Children's Hospital of Philadelphia, Philadelphia, PA 19104, USA; 2 Internal Medicine, University of Iowa, Iowa City, IA 52242, USA; 3 Division of Neuroscience, Oregon National Primate Research Center, Beaverton, OR 97006, USA; 4 Department of Behavioral Neuroscience, Oregon Health Sciences University, Portland, OR 97239, USA; 5 Department of Pathology and Laboratory Medicine, University of Pennsylvania, Philadelphia, PA 19104, USA

## Abstract

Huntington's disease is a fatal neurodegenerative disease caused by polyglutamine-expansion in huntingtin (HTT). Recent work showed that gene silencing approaches, including RNA interference (RNAi), improve disease readouts in mice. To advance RNAi to the clinic, we designed miHDS1, with robust knockdown of human HTT and minimized silencing of unintended transcripts. In *Rhesus macaque*, AAV delivery of miHDS1 to the putamen reduced *HTT* expression with no adverse effects on neurological status including fine and gross motor skills, no immune activation and no induction of neuropathology out to 6 weeks post injection. Others showed safety of a different HTT-targeting RNAi in monkeys for 6 months. Application of miHDS1 to Huntington's patients requires further safety testing in normal rodents, despite the fact that it was optimized for humans. To satisfy this regulatory requirement, we evaluated normal mice after AAV.miHDS1 injection. In contrast to monkeys, neurological deficits occurred acutely in mice brain and was attributed to off-target silencing through interactions of miHDS1 with the 3′UTR of other transcripts. While we resolved miHDS1 toxicity in mouse brain and maintained miHDS1-silencing efficacy, these studies highlight that optimizing nucleic acid-based medicines for safety in humans presents challenges for safety testing in rodents or other distantly related species.

## INTRODUCTION

Huntington's disease (HD) is a neurodegenerative disorder caused by CAG repeat expansion (>36 repeats) within the first exon of *huntingtin*. Although mutant huntingtin (mHTT) is ubiquitously expressed, the brain, and in particular the striatum, shows robust and early degeneration. The incidence of HD is ∼5–10 per 100 000 individuals in Europe and USA, with onset generally occurring in the 3rd or 4th decade of life. To date, management of HD is done pharmacologically to improve motor or psychiatric symptoms (1–3).

Earlier work with transgenic mice using a tetracycline-responsive system to express a pathogenic human huntingtin (HTT) fragment demonstrated that deleterious effects from the mutant protein, including the development of HTT-containing inclusions, reactive astrocytosis, decreases in dopamine D1 receptor levels, striatal atrophy and progressive motor symptoms, resolved with cessation of expression (4,5). These data imply that there is a window of opportunity to treat HD and support the notion that some symptoms may be reversible upon mutant HTT reduction. Therefore, the development of approaches to reduce gene expression using gene-silencing technologies, such us RNA interference (RNAi), hold great promise as a therapy for HD (6–9).RNAi is an evolutionarily conserved process of post-transcriptional gene silencing by which double-stranded small non-coding RNAs (e.g. miRNAs) cause sequence-specific degradation of target mRNA sequences. The endogenous RNAi pathway starts with the expression of a larger primary RNA transcript (pri-miRNA) that is sequentially cleaved in the nucleus by Drosha, a component of the microprocessor complex, to generate a precursor miRNA (pre-miRNA). Pre-miRNAs are exported to the cytoplasm and are subsequently cleaved by Dicer to release the mature miRNA. Generally, only one of the two strands (the antisense ‘guide’ strand) is incorporated preferentially into the RNA-induced silencing complex (RISC), where it directs binding to, and subsequent repression of, the target mRNA. Although endogenous miRNAs typically repress mRNA expression through partial complementarity, when a guide strand sequence is fully complementary to its target, the resulting small-interfering RNA (siRNA) directs endonucleotic cleavage of the target at a nucleotide paired to bases 10 and 11 of the ‘guide’ strand, triggering mRNA degradation (10). Scientists have developed various expression systems to co-opt the endogenous RNAi pathway and suppress the expression of specific genes. For example, RNAi vectors can be designed to express small hairpin RNA sequences entering the pathway at the pre-miRNA (short hairpin RNA; shRNA) or pri-miRNA (artificial miRNA) steps. Extensive work has been done to design RNAi sequences with high silencing efficacy for a given target (11–14).

For expression systems or siRNAs that are acutely transfected into cells, the active guide strand is designed to be as specific as possible with minimal off-sequence silencing. Off-sequence silencing arises from interaction of the guide strand with transcripts that are fully complementary. This type of off-targeting can be avoided using standard search algorithms. A more difficult type of off-targeting to avoid is that which occurs due to partial complementarity of the RNAi seed sequence (bases 2–7 at the 5′ end of the loaded strand) to other mRNA 3′UTRs. In this instance, repression of expression occurs via a miRNA-like mechanism (15). In previous studies, we developed an algorithm, siSPOTR, to design potent RNAi sequences with strong strand biasing for RISC loading, and minimized off-target silencing potential over unintended human transcripts (16). When siSPOTR was used to design triggers for human (HTT) and mouse huntingtin (Htt) silencing, we found that miHDS1, expressed from AAV vectors, showed safety in multiple assays ranging from animal behavior to neuronal health following delivery to non-human primate putamen (17). As a pre-requisite for human application, we performed subsequent experiments to evaluate miHDS1 tolerability in the mouse brain. Notably, we found that HDS1 induced acute motor deficits after striatal injections in mice. We surmised that this toxicity was due, at least in part, to unintended silencing. One putative target, *Bcl2* was validated as a direct target of HDS1. Importantly, *Bcl2* silencing via off-targeting could be resolved by several strategies while maintaining Htt-silencing efficacy. Overall these studies highlight the conundrum of optimizing nucleic acid-based medicines for specificity and safety in humans, but for which safety studies in rodents or other species are required. This is because distantly related species will portray different, and perhaps disease-inducing, off-targeting profiles.

## MATERIALS AND METHODS

### Sequence data

Human, rhesus and mouse 3′-UTR sequences and genomic coordinates were obtained from the UCSC Genome Browser hg19, rheMac3 and mm10 assemblies, respectively. Human and mouse 3′UTR sequences and coordinates were taken from RefSeq annotations. Off-target prediction was limited to protein-coding transcripts (NM prefix).

### Striatum expression

Genes expressed above background levels in mouse striatum were taken from RNA-seq measurements provided in Dataset S1 from Bottomly *et al*. (18).

### Off-target prediction

Off-target sites were predicted using the TargetScan6 Perl scripts (www.targetscan.org) using the default settings. Target sites and Context+ scores were calculated for human, rhesus and mouse 3′UTRs, ignoring sequence conservation. Genomic coordinates for the target sites were calculated, and redundant sites removed for each guide RNA.

Additional thermodynamic information was assessed for a subset of TargetScan-predicted off-targets using the PITA target prediction algorithm (http://genie.weizmann.ac.il/pubs/mir07/mir07_exe.html). PITA was run using relaxed seed-match parameters (-gu ‘6;0,7;1,8;1’ and –mm ‘6;0,7;1,8;1’). Genomic coordinates were calculated for the PITA sites, and were used to intersect with the TargetScan predictions. PITA sites without a corresponding TargetScan prediction were removed.

#### Off-target conservation analysis between human, mouse and rhesus

Sequences and genomic coordinates for Ensembl-annotated human (hg19) and mouse (mm9) 3′UTRs were downloaded using the UCSC Genome Table Browser tool. Rhesus 3′UTR sequences and genomic coordinates (rheMac3) were extracted based on synteny with the aforementioned human coordinates. Here, human 3′UTR coordinates were converted to rhesus genomic coordinates using the liftOver tool available from UCSC Genome Browser tools, setting the minimum ration of bases that remap to 0.1 and the minimum matching region size at 30 bases.

TargetScan6.0 was used to predict miHDS1 off-targets in the human, mouse and rhesus 3′UTRs as described above. An off-target was considered ‘conserved’ between any two of the species if at least one target site was found anywhere in orthologous gene's 3′UTR. Human Ensembl gene IDs were retained in the conversion to rhesus coordinates, and were used to determine orthology. High-confidence human and mouse ortholog annotations were obtained from Biomart. Assessment of conserved off-targeting was limited to these genes. Off-target genes were annotated and grouped based on the human Ensembl gene IDs, calculating the minimum (strongest) context+ score, where present.

### Cell lines and transfections

HEK293 were obtained from ATCC and cultured under conditions provided by manufacturer. SthdhQ7 were kindly obtained from Marcy MacDonald (19). All plasmid DNA transfections on HEK293 were done with Lipofectamine 2000 (Invitrogen) using guidelines provided by manufacturer. DNA transfection of SthdhQ7 cells was done using a Invitrogen Neon transfection system using the electroporation conditions (1350 V, 30 ms, 1 pulse) and following the guidelines provided by manufacturer.

### Vector design and AAV production

Artificial miRNA sequences (miCtl, miHDss variants, miHDS1 and miHDS1 variants) were generated by polymerase extension of overlapping DNA oligonucleotides (IDT, Coralville). Polymerase-extended products were purified using Qiaquick PCR purification kit, digested with XhoI-SpeI and cloned into a XhoI-XbaI site on a Pol-III expression cassette containing the mouse U6 promoter, a multiple cloning site and the Pol-III-terminator (6T's) (20). DNA oligonucleotides used for artificial miRNA generation are listed in Supplementary Table S5.RNAi luciferase reporter vectors were constructed in psiCheck2 vector (Promega). Tailed DNA oligonucleotides containing a single, perfect complementary RNAi target site for miHDS1 sense or antisense strands were annealed and cloned into an XhoI-NotI sites downstream of the stop codon of the *Renilla* luciferase cDNA sequence. Tailed primer pairs used to generate luciferase reporters are listed in Supplementary Table S5.

For *in vivo* studies, miRNA expression cassettes were moved into an AAV shuttle plasmid upstream of a DNA stuffer sequence. The stuffer sequence was obtained by amplification and assembly of intronic sequences of human HTT and was designed to be devoid of enhancer or repressor sequences, splice activators or repressors, and antisense or other non-coding RNAs. The artificial miRNA expression cassette and stuffer sequence were flanked at each end by AAV serotype 2 145-bp inverted terminal repeat sequences.

### *In vitro* luciferase assays

HEK293 cells at 70% confluence in a 24-well plate were co-transfected with miRNA-expressing plasmids and RNAi luciferase reporter plasmids. At 24 h, cells were rinsed with ice-cold phosphate-buffered saline (PBS) and *Renilla* and *Firerfly* luciferase activities were assessed using the Dual-Luciferase Reporter Assay System (Promega) according to manufacture's instructions, using 20 μl of cell lysate. Luminescent readouts were obtained with a Monolight 3010 luminometer (Pharmigen, USA). Relative light units were calculated as the quotient of *Renilla/Firefly* relative light units and results expressed relative to a control miRNA.

### Western blot analysis

HEK293 cells were transfected with miRNA expression cassettes as indicated. At 48 h cells were rinsed once with iced-cold PBS and lysed with Passive lysis buffer (PBL, Promega). Protein concentration was determined by the Bradford–Lowry method (BioRad) and 10 μg of protein loaded on a NuPAGE 3–8% Tris-Acetate gel (Novex Life Technologies). Proteins were transferred onto polyvinylidene fluoride (PVDF) membranes and incubated with a mouse anti-Htt (1:5000, Millipore, CA, USA), or rabbit anti-Beta-actin (1:40000, Sigma) antibodies followed by horseradish peroxidase-coupled antibodies (1:10,000, mouse; or 1:50,000, Rabbit; Jackson ImmunoResearch, West Grove, PA, USA). Blots were developed with ECL-Plus reagents (Amersham Pharmacia). Silencing efficacy was determined by densitometry (*n* = 4 independent experiments) of protein levels relative to beta actin with the VersaDoc^TM^ Imaging System (Biorad) and Quantity One^R^ analysis software.

### RNA extraction and reverse transcriptase-quantitative polymerase chain reaction analysis

Total RNA was extracted using Trizol (Life Technologies, Grand Island, NY, USA) according to the manufacturer's protocol, with the exception of 1 μl Glycoblue (Life Technologies, Grand Island, NY, USA) in addition to the aqueous phase on the isopropanol precipitation step and a single wash with cold 70% ethanol. RNA samples were quantified by spectrophotometry and subsequently cDNAs generated from 500 ng of total RNA with random hexamers (TaqMan RT reagents, Applied Biosystems). SyBrGreen reverse transcriptase-quantitative polymerase chain reaction (RT-qPCR) primer pairs for mouse off-target genes were designed using the RealTime PCR Custom Assay Design webserver (IDT, Coralville). A seven-point standard curve with a final melting curve assay was performed to validate each primer pair. Only primer pairs with amplification efficiencies of a 100 ± 5% and a single amplification product were used to determine relative gene expression using the ΔΔCt method. miRNA expression levels in mouse striatum were determined using a stem-loop RT-PCR reaction as described in (21) with some modifications. Briefly, each RT reaction included a miRNA stem loop RT-PCR primer mixed with a specific mouse U6snRNA RT-primer as a control. Next, miRNA and U6 snRNA transcripts were quantified using 1 μl of RT product by semi-quantitative PCR (30 cycles, miRNA sequences; 25 cycles, mU6snRNA). PCR products were separated by electrophoresis on a 3% agarose gel stained with ethidium bromide, and DNA bands were quantified using Image Lab 5.1 software (Biorad). Primer pairs used for real-time and semi-quantitative PCR are listed in Supplementary Table S5.

### Mouse studies

All animal protocols were approved by the University of Iowa Animal Care and Use Committee. Wild-type FBV mice were obtained from Jackson Laboratories (Bar Harbor, ME, USA). Mice were housed in a temperature-controlled environment on a 12-h light/dark cycle. Food and water were provided ad libitum. At the indicated times, mice were injected with AAV2/1-mU6-miRNA/Stuffer virus. For AAV injections, mice were anesthetized with a ketamine and xylazine mix, and 5 μl of AAV injected bilaterally into striata at 0.2 μl/min (coordinates: +0.86 mm rostral to Bregma, +/−1.8 mm lateral to medial, −2.5 mm ventral from brain surface). Mice used for gene expression analyses were anesthetized with a ketamine and xylazine mix and perfused with 18 ml of 0.9% cold saline mixed with 2-ml RNAlater (Ambion) solution. At the indicated times, mice were sacrificed and the brain was removed, blocked and cut into 1-mm-thick coronal slices. Tissue punches from striata were taken using a tissue corer (1.4-mm in diameter; Zivic Instruments, Pittsburgh, PA, USA). All tissue punches were flash frozen in liquid nitrogen and stored at −80C until use.

### Behavior analysis

Motor coordination of injected mice was determined using the Rotarod apparatus (model 47,600; Ugo Basile, Comerio, Italy). A basal rotarod test was performed at 7 weeks of age and again 2 and 4 months after AAV injection. Mice were tested for four consecutive days with three trials per day, with a 30-min period of rest between trials and a 5-min habituation period each day beginning 60 min before the first trial. The latency to fall per mouse was calculated by averaging two trials per day per mouse over the four consecutive days tested.

### Statistical analysis

All statistical analyses were performed using GraphPad Prism v5.0 software. Data with normal distributions (passed D'Agostino normality test) were analyzed using an unpaired *t*-test, or one-way ANOVA followed by a Bonferroni's post-hoc. Otherwise, data without normal distribution were analyzed using a Mann–Whitney test, or Kruskal–Wallis test followed by a Dunn's post-hoc. In all cases, *P* < 0.05 was considered significant.

## RESULTS

### miHDS1 induces neurological deficits in the mouse brain

**Figure 1.**
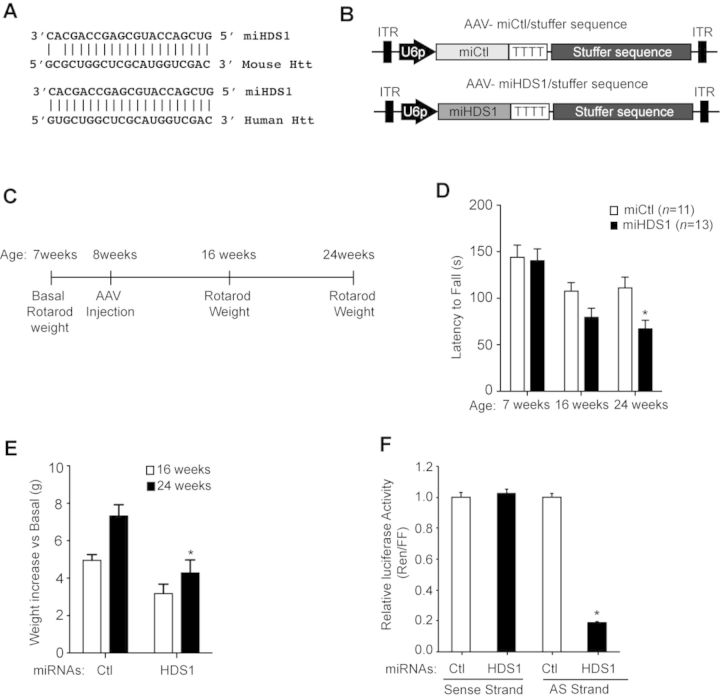
Overexpression of miHDS1 causes adverse effects in the mouse brain. (**A**) miHDS1 pairing to mouse and human huntingtin mRNA. (**B**) Cartoon depicting AAV/stuffer shuttle vectors containing miHDS1 and miCtl expression cassettes. (**C**) Experimental strategy to evaluate miHDS1 *in vivo* tolerability. (**D**) Rotarod data from mice injected with miHDS1 (*n* = 13) or miCtl (*n* = 11). Latency to fall is shown as mean ± SEM. (*P* > 0.05, unpaired *t*-test at the indicated times). (**E**) Weight gain analysis of mice injected with miHDS1 and miCtl. Data are shown as weight gain with respect to baseline. (**F**) Strand biasing of the U6/miHDS1vector. Strand biasing was assessed measuring luciferase activity from reporter constructs containing target sequences complementary to the passenger (sense) or guide (antisense) miHDS1 strands. Results are representative experiment of three different experiments (*n* = 4/group). Data are shown as mean ± SEM relative to cells transfected with miCtl and demonstrate that miHDS1 preferentially loads the guide miHDS1 strand.

In prior work we designed miHDS1, an artificial miRNA sequence targeting huntingtin with a low silencing potential over unintended human transcripts (Figure 1A) (11). When AAV vectors expressing miHDS1 were injected into the putamen of non-human primates (NHPs), HTT levels were significantly reduced and there were no signs of neuronal degeneration, immune responses or motor deficits (17). Overall, these studies highlighted the potential of miHDS1 for HD therapeutics. However, as a pre-requisite for human application, further testing in another species, such as rodents, is required. Thus we set out to perform safety testing of AAV.miHSD1 in normal mice, despite the fact that it was designed for safety in human cells.

As a first step in building the preclinical construct, we redesigned the AAV.miHSD1 vector to contain a stuffer sequence rather than the eGFP expression cassette, which was used in our earlier studies for visualization of transduced regions. The stuffer sequence was designed to be devoid of enhancer or repressor sequences, splice activators or repressors, and antisense or other non-coding RNAs, and of sufficient size for optimal packaging of the small RNAi expression cassette into AAV capsids. The final AAV2/1 vectors expressed miHDS1 or miCtl, a control used in our previous *in vivo* studies (22,23) (Figure 1B).

Wild-type mice were weighed and basal rotarod performance assessed at 7 weeks of age to distribute animals into groups of equal abilities (to avoid pre-treatment differences between the groups). AAV.miHDS1 or AAV.miCtl were injected bilaterally into the striatum at 8 weeks of age with AAVmiHDS1/Stuffer (*n* = 13) and AAVmiCtl/Stuffer (*n* = 11) virus (Figure 1B and C). As early as 2 months after AAV delivery, mice expressing miHDS1 had significant rotarod deficits and showed decreased latency to fall with respect to control-treated littermates (Figure 1D). All animals gained weight over the course of the study; however, HDS1-treated mice gained significantly less weight than miCtl-treated mice (Figure 1E). In similar studies, miHDS1 treatment of HD mice decreased latency to fall on the rotarod and caused weight loss (Supplementary Figure S1). Taken together, these data demonstrate that miHDS1 expression in the mouse striatum is not well tolerated.

### Characterization of miHDS1 off-target genes in the mouse brain

Because no overt toxicity was observed in human cell lines or in rhesus brains, and miHDS1 was designed with a minimized number of off-target genes in several species (human, rhesus and mouse), we were surprised to find such robust toxicity in the mouse brain. Although the AAV.miHDS1.eGFP construct used earlier in NHPs showed appropriate strand loading, we first tested the fidelity of the miHDS1.stuffer expression cassette for strand biasing, as either strand, if loaded, could illicit off-target silencing. For this we designed reporter constructs consisting of miHDS1 targets cloned downstream of a luciferase reporter. We found repression from the guide strand, and no repression from the non-guide strand (Figure 1F). This is in line with our earlier *in vitro* expression analyses of miHDS1.eGFP expression cassettes, and is supported by the fact that we designed the miHDS1 sequence with low 5′ end thermodynamic stability to promote proper loading of the guide ‘antisense’ strand into the RISC complex (24). Thus, the neuronal deficits observed by miHDS1 expression are likely due to the binding of the guide ‘antisense’ strand to the 3′UTR of unintended mRNAs and silencing expression by a miRNA-like mechanism (15).

**Figure 2.**
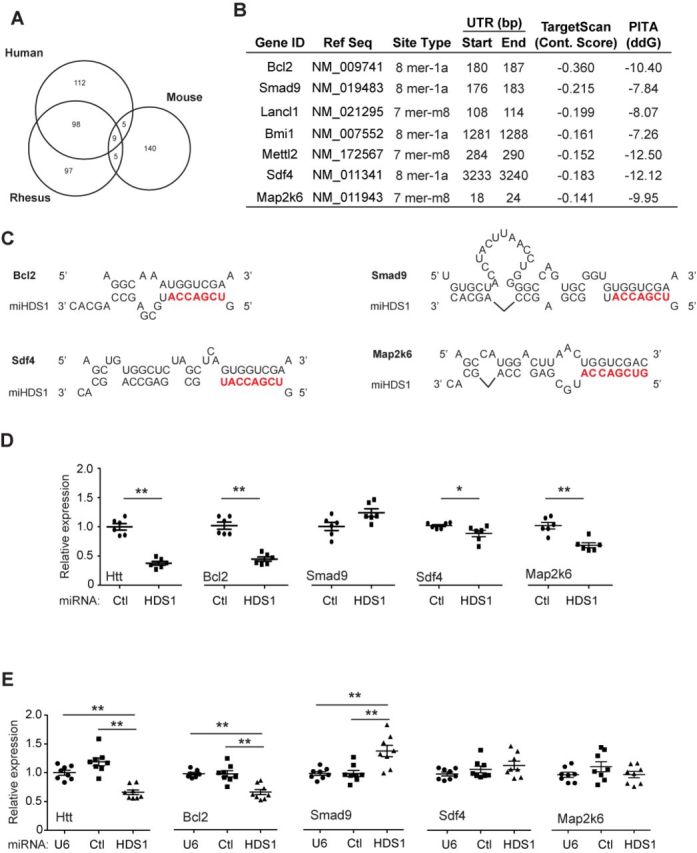
Characterization of miHDS1 off-target genes. (**A**) miHDS1 off-target conservation between human, rhesus and mouse. (**B**) List of miHDS1 off-target genes tested. Information displayed: gene ID, reference sequence, miRNA-binding site type, nucleotide 3′UTR position, predicted target scan context score, ΔΔ*G* score predicted by PITA algorithm. (**C**) Cartoon depicting miHDS1:mRNA-binding sites on predicted off-targeted genes. (**D**) RT-qPCR analysis of *Htt*, *Bcl2*, *Smad9*, *Sdf4* and *Map2k6* mRNA levels in striatum samples 4 months after miHDS1 injection. All samples were normalized to *ß-actin* and results are the mean ± SEM relative to mice injected with miCtl. (*n* = 6 mice per group; *P < 0.05, **P < 0.01, Mann–Whitney test) (**E**) RT-qPCR analysis of *Htt*, *Bcl2*, *Smad9*, *Sdf4* and *Map2k6* mRNA levels in SthdhQ7 cells after miHDS1 electroporation. All samples were normalized to *ß-actin* and results are the mean ± SEM relative to cells electroporated with plasmids containing a non-expression control plasmid (U6 promoter only) or miCtl expression cassettes. (*n* = 8 electroporated wells; **P < 0.01, one-way ANOVA followed by a Bonferroni's post-hoc).

**Figure 3.**
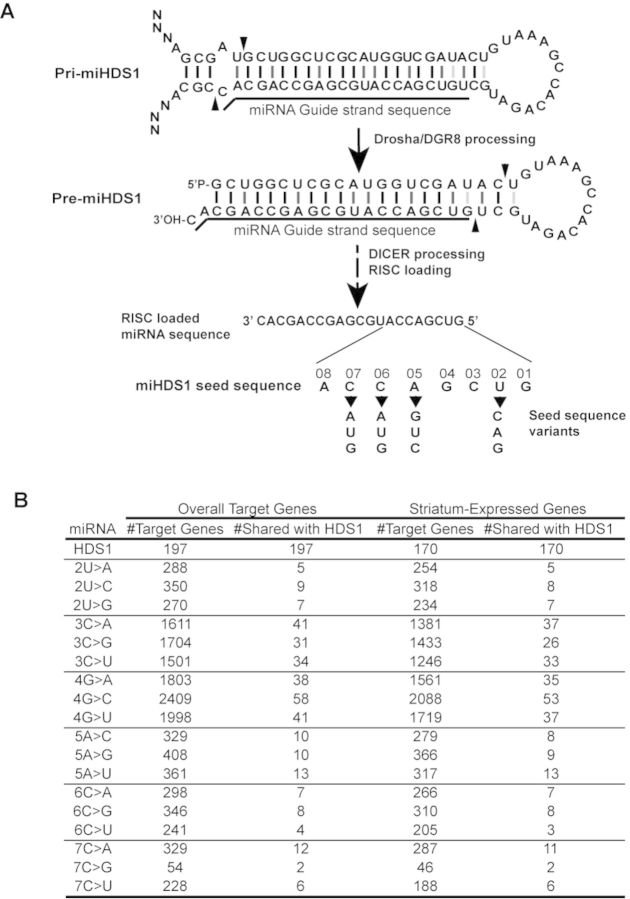
Generation of single nucleotide miHDS1 seed variants. (**A**) Cartoon depicting location of single nucleotide modifications in the seed region of miHDS1 sequence. (**B**) Table indicates the number of predicted off-target genes (overall and striatum-specific) for miHDS1 and miHDS1 variants, as well as the number of shared off-targets.

Because previous studies demonstrated that most off-target effects are due to seed-mediated binding to other mRNA 3′UTRs, we first identified likely mouse miHDS1 off-targets using a common *in silico* approach. Many different target prediction programs have been described to identify putative miRNA-binding sites, including the TargetScan (TS) and PITA algorithms (25). TargetScan predicts biological targets for a specific miRNA by searching 3′UTR sequences for the presence of 8mer and 7mer sites complementary to the miRNA seed sequence. The algorithm improves target prediction accuracy by prioritizing target sites with compensatory 3′ base pairing, local sequence context and strong sequence conservation known to be favorable for miRNA-mediated regulation (26,27). Because previous work has shown that siRNA seed-mediated off-target effects are species-specific, we used TargetScan to predict targets based on seed sequence complementarity in the mouse 3′UTR transcriptome (28). The PITA algorithm incorporates target-site accessibility to predict miRNA-binding sites (29). For a given target site, PITA determines a ΔΔ*G* value, which represents the minimum free energy difference between the miRNA:target hybridization (Δ*G*_duplex_) and local secondary structure of the target site (Δ*G*_open_). Based on PITA documentation, ΔΔ*G* scores below −10 kcal/mol are more likely to be functional for endogenous miRNA targets, although the threshold for an overexpressed miRNA sequence could be higher (>−10 kcal/mol) (29). Thus, in our approach we used TargetScan to identify all potential seed-binding sites, followed by the PITA algorithm to determine the ΔΔG score, and used this information to rank potential miHDS1 sites. Using our approach against the mouse 3′UTRs, we predict 197 transcripts as potential miHDS1 off-targets, with 170 expressed in the striatum (Figure 3B) (18). Off-targets in the orthologous human and rhesus 3′UTRs revealed that the miHDS1 off-targeting in mouse is not conserved (Supplementary Table S1, Figure 2A). This is not unexpected because there is little conservation between the 3′UTRs of distant species (28). Hence, there exists the possibility of hitting one or more critical gene products in a species-specific manner.

We identified *Bcl2*, *Sdf4*, *Smad9*, *Bmi1*, *Mettl2*, *Lancl1* and *Map2k6* among the leading off-target gene list (Figure 2B and C). We analyzed striatal samples obtained from mice treated with miCtl or miHDS1 by RT-qPCR for these predicted off-targets as well as confirmed mouse Htt knockdown. As expected, mouse Htt expression was significantly reduced (up to 70%) in miHDS1-treated mice as compared to mice treated with miCtl (Figure 2D). Among the set of off-target transcripts assessed, *Bcl2*, *Sdf4* and *Map2k6* were significantly reduced in tissue samples obtained from mice treated with AAV.miHDS1 (Figure 2D). No off-target sites for miCtl were predicted in these transcripts (Supplementary Table S2).

We confirmed these results *in vitro* using an immortalized mouse neuronal striatal cell line that has a normal Htt allele (SthdhQ7 cells). SthdhQ7 cells were electroporated with plasmids expressing miHDS1, miCtl or no transcript (plasmid contained only the U6 promoter), and 24 h later transcripts were analyzed by Q-PCR. As observed in mouse brain, *Bcl2* expression was reduced in miHDS1-expressing cells, but not those expressing miCtl or treated with a non-expression control plasmid (Figure 2D and E). In contrast, *Sdf4* and *Map2k6* expression was not reduced by overexpression of miHDS1 (Figure 2D and E), suggesting that these genes may not be direct off-targets *in vivo*, and may reflect indirect effects of Htt suppression over time or off-target suppression in non-neuronal cells, although AAV2/1 transduces primarily neurons (30,31). Interestingly, *Smad9* expression was significantly increased in SthdhQ7 cells, and was elevated, though not significantly so, in miHDS1-treated striata (Figure 2D and E). Thus, our screen revealed *Bcl2* as a potential deleterious off-target of HDS1 in the mice 3′ UTRome.

### Modifying miHDS1 for safety in mouse brain

When a miRNA sequence is loaded into RISC containing a catalytic argonaute protein (Ago2), full binding complementarity between a miRNA and its target sequence is generally required to mediate endonucleotic mRNA cleavage. However, mismatches produced by single point mutations on the miRNA sequence can be tolerated, given that extensive central pairing between the miRNA and its target is retained (32–34). Thus, to modify the off-target profile of miHDS1, which is directed primarily by the seed region, we introduced single point mutations that were designed to alter the seed without affecting silencing efficacy (Figure 3A).

As a first step to identify seed mutations which (i) effectively change the off-target profile, (ii) maintain low overall off-targeting potential and (iii) silence human HTT, we repeated the off-target prediction analysis using all single nucleotide seed variants (positions 2–7) of miHDS1 (Figure 3B). Position 8 mutants were discarded, because the off-target profile extensively overlapped that of miHDS1. This was expected, since position 8 pairing is not necessary for miRNA-mediated silencing (35). Seed mutants at positions 3 and 4 were also discarded, since these mutations significantly increased the number of predicted off-targets. This was also expected, because these mutations disrupt a ‘CG’ dinucleotide, which are rare in mammalian 3′UTRs (16,36). For the remaining seed variants, overall off-targeting potential was comparable to miHDS1, with less than 10% of miHDS1 off-targets being shared with the miHDS1 variants (Figure 3B).

**Figure 4.**
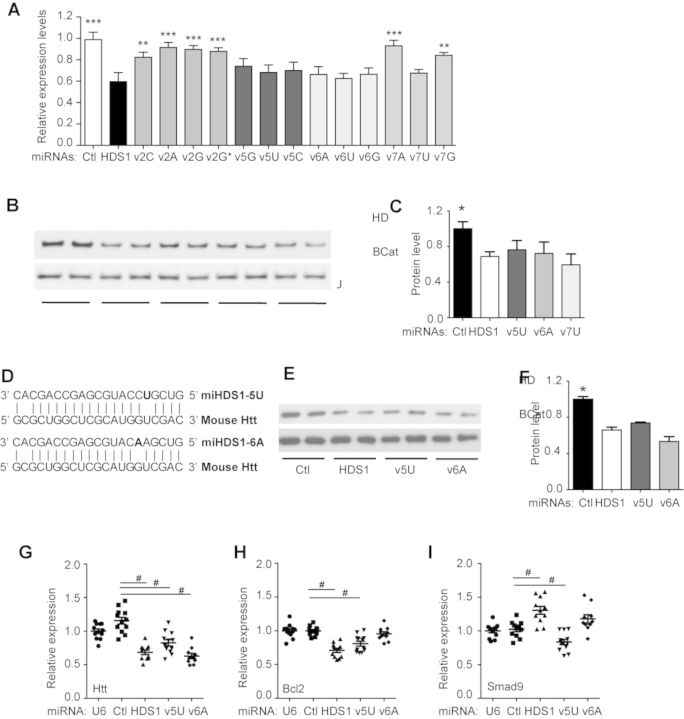
Silencing efficacy of single nucleotide miHDS1 seed variants on Htt expression. (**A**) Quantitative analysis of HTT mRNA levels in HEK293 cells transfected with U6/miHDS1 expression cassettes. Total RNA was collected 24 h post-transfection and HTT transcripts were determined by RT-qPCR. All samples were normalized to *ß-actin* and results are the mean ± SEM relative to cells transfected with miCtl (*n* = 12 wells; **P < 0.01, ***P < 0.001, constructs were tested for statistical difference to miHDS1 by one-way ANOVA followed by a Bonferroni's post-hoc). (**B**) miHDS1, miHDS1v5U, miHDS1v6A and miHDS1v7U expression cassettes were transfected into human HEK293 cells, and endogenous HTT protein levels were determined 48 h after transfection. miCtl was used as a no silencing control and β-Catenin serves as a loading control. (**C**) Quantification of HTT protein levels 48 h after transfection of miHDS1, miHDS1v5U, miHDS1v6A and miHDS1v7u. Data are the mean ± SEM relative to cells transfected with miCtl (*n* = 6, three different western blots, *P < 0.01, constructs were tested for statistical difference to miHDS1 by Mann–Whitney test). (**D**) miHDS1v5U and miHDS1v6A pairing to mouse huntingtin mRNA. (**E**) miHDS1, miHDS1v5U and miHDS1v6A expression cassettes were electroporated into mouse SthdhQ7 cells, and endogenous Htt protein levels were determined 48 h after electroporation. miCtl was used as a no-silencing control and β-Catenin serves as a loading control. (**F**) Quantification of mHtt protein levels 48 h after electroporation of miHDS1, miHDS1v5U and miHDS1v6A. Data are the mean ± SEM relative to cells transfected with miCtl (*n* = 6, three different western blots, *P < 0.01, constructs were tested for statistical difference to miHDS1 by Mann–Whitney test). (**G**–**I**) RT-qPCR analysis of *mHtt*, *Bcl2* and *Smad9* mRNA levels in SthdhQ7 cells electroporated with U6/miHDS1, U6/miHDS1v5U and U6/miHDS1v6A expression cassettes. Total RNA was collected 24 h post-electroporation and transcript levels were determined by RT-qPCR. All samples were normalized to *ß-actin* and results are the mean ± SEM relative to cells transfected with U6 containing promoter and U6/miCtl expression cassette (*n* = 12 wells; # *P* < 0.01, one-way ANOVA followed by Bonferroni's post-hoc).

We next introduced single point mutations at positions 2, 5, 6 and 7 of the miHDS1 seed region to generate the miHDS1 variants. Because our goal is silencing of human HTT, we first screened all variants in human-derived HEK293 cells and determined silencing efficacy by RT-qPCR (Figure 4A). Not all miHDS1 variants reduced Htt expression equivalent to the original miHDS1. Compared to miHDS1, mHDS1 variants with mismatches at positions 2 and 7 disrupt miHDS1-silencing efficacy. However, no significant differences were observed for miHDS1 and those variants containing a mismatch at position 5 or 6. Of note, among the different miHDS1 variants with a mismatch at position 7 only the variant with a C>U substitution had equivalent silencing efficacy to miHDS1, presumably due to the thermodynamic stability of the G:U wobble. Thus, miHDS1v6A and miHDS1v5U were used for further experiments based on: (i) the higher silencing efficacy with respect to the other miRNA variants containing a mismatch at the same seed position, and (ii) the nucleotide mismatch type generated (U:U, miHDS1v5U; A:G, miHDS1v6A, Figure 4D) maintains an off-target profile that differs extensively from HDS1 (Figure 3B). As expected, expression of miHDS1v6A and miHDS1v5U reduced Htt protein levels in both mouse (SthdhQ7) and human (HEK293) cells, similar to miHDS1 (Figure 4B, C, E and F).

Next we evaluated miHDS1 off-target transcripts after miHDS1v6A and miHDS1v5U expression. TargetScan did not recognize any of these target sites for miHDS1 as predicted off-target sites for the miHDS1 variants. However, the possibility exists that 3′ compensatory interactions could minimize the effect of single nucleotide mismatches in the seed. Using PITA to determine the ΔΔG score for both miHDS1v6A and miHDS1v5U on the predicted miHDS1 sites showed that the introduction of a seed mismatch reduced the ΔΔG value for all predicted off-target genes, being more significant for miHDS1v6A (6.6 kcal/mol) than miHDS1v5U (3.9 kcal/mol).

Based on TargetScan, miHDS1v6A and miHDS1v5U will no longer target the *Bcl2* 3′UTR, and PITA predicts a reduced ΔΔG score at the miHDS1 site (miHDS1:−10.4, miHDS1v6A: −3.8 kcal/mol, miHDS1v5U: −6.5 kcal/mol), suggesting that *Bcl2* silencing will be less effective. To test this, SthdhQ7 cells were electroporated with plasmids containing the miRNA expression cassettes or the control plasmid, and 24 h later *Bcl2*, *Htt* and *Smad9* expression determined by Q-PCR. Relative to controls (miCtl and U6), *Htt* mRNA levels were significantly reduced in miHDS1 and miHDS1-variant electroporated cells (Figure 4G). miHDS1 reduced *Bcl2* expression by 40%, as before, but there was no observed silencing after electroporation of miHDS1v6A. MiHDS1v5U was still active against *Bcl2*, silencing 20% compared to control-treated cells (Figure 4H). Interestingly, Smad9 overexpression associated with miHDS1 expression was not observed in miHDS1v5U or miHDS1v6A-electroporated cells (Figure 4I).

### Redirecting miCtl against human huntingtin mRNA

Our previous experiments exposed the toxicity of miHDS1 due to its off-target effects, but also highlighted that miCtl is tolerable when expressed in the mouse striatum. miCtl was designed with a low off-target silencing profile, but was not intended to target the huntingtin mRNA. Therefore, we tested if we could take advantage of the relative safety of the miCtl seed in mouse striata, and design a HTT-targeting RNAi trigger around that seed.

**Figure 5.**
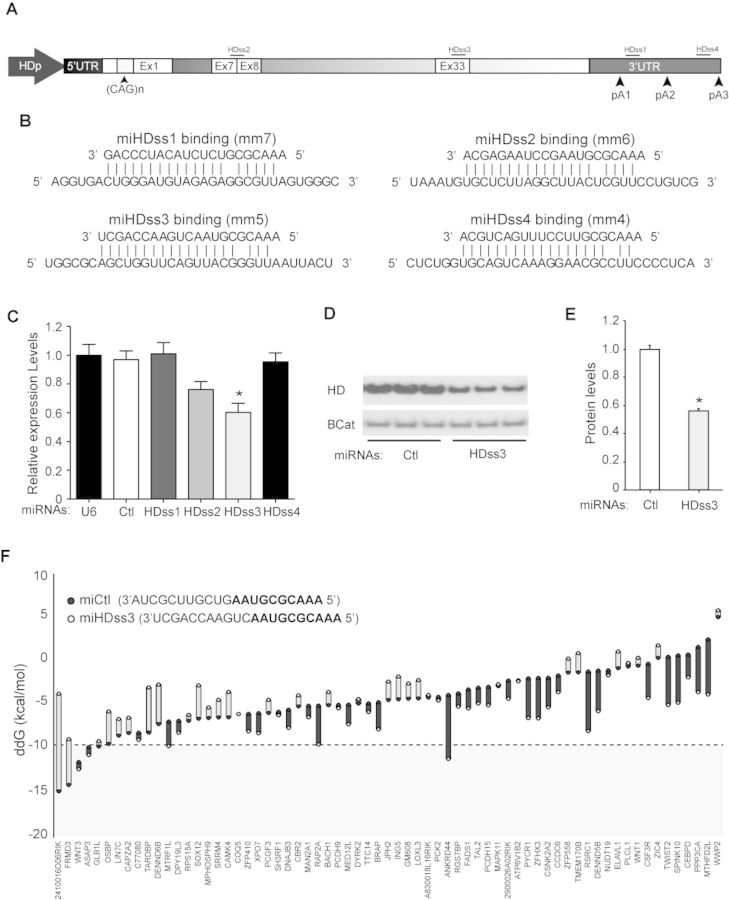
Generation of miHDss1–4 sequences to target human *HTT* expression. (**A**) Four artificial miRNA triggers containing miCtl seed sequence were generated allowing a single nucleotide mismatch between seed region and targeted human *HTT* mRNA. miHDss1 and miHDss4-binding sites are located at the 3′UTR, whereas miHDss2 and 3 bind at exon 7–8 boundary and exon 33 of *HTT*, respectively. (**B**) miRNA/mRNA binding between miHDss1–4 and human huntingtin mRNA. Single nucleotide mismatches are at seed region positions 7, 6, 5 and 4 for miHDss1, 2, 3 and 4 sequences, respectively. (**C**) Quantitative analysis of *HTT* mRNA levels in HEK293 cells transfected with U6/miHDss1–4 expression cassettes. Total RNA was collected 24 h post-transfection and transcript levels determined by RT-qPCR. All samples were normalized to *ß-actin* and results are the mean ± SEM relative to cells transfected with miCtl (*n* = 8 wells; *P < 0.001, one-way ANOVA followed by a Bonferroni's post-hoc). (**D**) miHDss3 expression cassette was transfected into human HEK293 cells, and endogenous HTT protein levels were determined 48 h later. miCtl was used as a negative control and β-Catenin serves as a loading control. (**E**) Quantification of HTT protein levels 48 h after transfection of miHDss3. Data are the mean ± SEM relative to cells transfected with miCtl (*n* = 6, *P < 0.01, Mann–Whitney test). (**F**) The PITA algorithm was used to determine binding stability of miHDss3 and miCtl over predicted unintended mRNA binding sites. Seed region of miCtl and miHDss3 are highlighted in bold. Data are shown as a ΔΔG (kcal/mol) score for each off-target gene with respect to miCtl or miHDss3.

As a first step, we screened the human *HTT* mRNA, our clinical target, for sequences fully complementary to the miCtl seed region, but found none. Following the same strategy for designing miHDS1 variants, we repeated this *in silico* analysis allowing single mismatches between nucleotides 2 to 7 of the miRNA seed sequence. We found four complementary sequences (miHDss1–4): MiHDss1 and miHDss4 target HTT in the 3′UTR, whereas miHDss2 and miHDss3 target HTT in the coding region spanning the exon 7–8 juncture or in exon 33, respectively (Figure 5A and B). When tested in HEK 293 cells, only miHDss3 silenced HTT expression 40–50% relative to control-treated cells, as determined by RT-qPCR (Figure 5C) and western blot (Figure 5D and E).

Because miCtl and miHDss3 share the same seed sequence, we expect that both miRNAs will have the same off-target profile. However, as observed for endogenous miRNAs, silencing efficacy might change because of sequence differences on the 3′ region. We used the PITA algorithm to compare miRNA-binding stability and silencing potential between miCtl and miHDss3 off-targets. Our *in silico* approach predicts 89 off-target sites for miCtl and miHDss3, with 67 expressed in striatum (Supplementary Tables S2 and S4). Interestingly, on average, the 3′ region of miHDss3 increases off-target-miRNA-binding stability as compared with miCtl (Figure 5F).

### Characterization of miHDS1 variants and miHDss3 tolerability in the mouse brain

**Figure 6.**
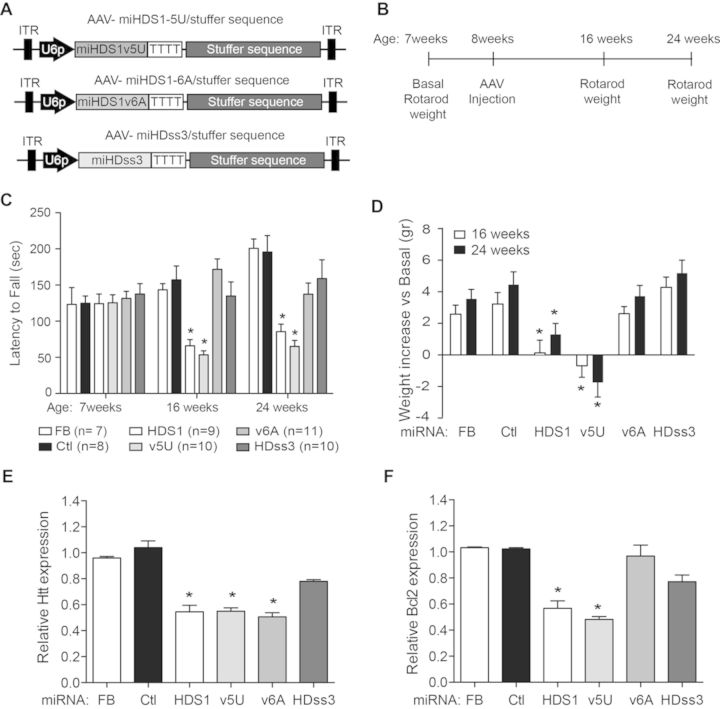
*In vivo* tolerability of miHDS1 variants and miHDss3 sequences. (**A**) Experimental strategy to evaluate *in vivo* tolerability of the new miRNA sequence design. (**B**) Cartoon depicting AAV/stuffer shuttle vectors containing miHDS1 variants and miHDss3 expression cassettes. (**C**) Rotarod data from mice injected with FB (*n* = 7), miCtl (*n* = 8), miHDS1 (*n* = 9), miHDS1v5U (*n* = 10), miHDS1v6A (*n* = 11) or miHDss3 (*n* = 10). Latency to fall is shown as mean ± SEM relative to mice injected with miCtl. (*P < 0.05, one-way ANOVA followed by Bonferroni's post-hoc). (**D**) Mice weights following injection of FB, miCtl, miHDS1, miHDS1v5U, miHDS1v6A or miHDss3. Data are shown as increase in weight with respect to baseline (*P < 0.05, one-way ANOVA followed by a Bonferroni's post-hoc). (**E**) Silencing of *Htt* by the miRNA expression cassettes (RT-qPCR) (*P < 0.05, Kruskal–Wallis followed by a Dunn's post-hoc). (**F**) Silencing of *Bcl2* by the miRNA expression cassettes (RT-qPCR) (*P < 0.05, Kruskal–Wallis followed by a Dunn's post-test).

To determine the *in vivo* tolerability of the new sequences, the miRNA expression cassettes were cloned into our AAV shuttle vector (Figure 6A). Seven-week old wild-type mice were divided into groups based on equivalent weight and basal rotarod performance, and subsequently injected bilaterally in the striatum with virus expressing miHDS1v6A, miHDS1v5U, miHDss3 or miHDS1. MiCtl or formulation buffer (FB) was used as negative experimental control. Two and four months after injection mouse weight was recorded, and adverse neurologic effects were determined using the accelerated rotarod test (Figure 6B).

Consistent with our previous results (Figure 1D), mice expressing miHDS1 showed motor deficits on the accelerated rotarod apparatus (Figure 6C). Also, no differences were observed between mice injected with FB buffer alone or miCtl. This result is important because it suggests that adverse effects are not due to co-opting the endogenous pathway, but to specific miHDS1 off-target effects. Interestingly, and consistent with our *in vitro* studies, miHDS1v5U showed rotarod deficits as well. This may reflect that pyrimidine:pyrimidine mismatches (U:U, miRNA:mRNA) display moderate discrimination power and this variant still partially silenced *Bcl2* (Figure 4H). Alternatively, this miRNA could be reducing the expression of yet a different gene that imparts some toxicity. Also predicted from our *in vitro* work, miHDS1v6A improved miHDS1-mediated toxicity, with no significant differences observed between miHDS1v6A and miCtl at 2 or 4 months after AAV injection. No significant differences in accelerating rotarod performance were observed between miCtl and miHDss3-treated mice at 2 or 4 months post-injection (Figure 6C). Besides silencing human huntingtin, miHDss3 shares the same off-target profile than miCtl. However, the PITA algorithm suggested miHDss3 is more prone to silence the miCtl off-target repertoire by increasing the binding stability of the miRNA:mRNA pair (Figure 5F). However, no significant differences were observed on the accelerating rotarod at 2 or 4 months (Figure 6C).

With the exception of mice injected with miHDS1v5U that lost weight over time (−1.7 g, 8% reduction at 4 months), body weight gain was observed in all other groups. Also, weight gain was significantly reduced with miHDS1 treatment, as seen previously (Figure 1E). At 4 months, miHDS1-injected mice had 1.3 g (5%) of body weight gain, whereas the other groups had weight increases from 3.6 and 5.2 g (15–22% increase) (Figure 6D).

Brain transcripts were analyzed to confirm miRNA expression and wild-type huntingtin silencing *in vivo*. We found no correlation between toxicity and relative expression levels (Supplementary Figure S2). miHDS1 and the single nucleotide seed variant miHDS1v5U expression were not significantly different than other miRNA sequences, and silenced Htt to similar levels, yet had variable toxicity *in vivo*. MiHDss3 was designed to target human but not mouse Htt mRNA. As expected, there were no significant differences on huntingtin expression between mice expressing miCtl and miHDss3 (Figure 6E). Next, we evaluated *Bcl2* expression in all injected mice to determine if off-target silencing correlated with the adverse neurological effects. Relative to miCtl and FB controls, *Bcl2* expression was significantly reduced in mice expressing miHDS1 and miHDS1v5U, but not in mice expressing miHDS1v6A or miHDss3 (Figure 6F). These data demonstrate that adverse neurological effects are related to miHDS1 off-targeting, but not to reduced mouse Htt levels. Cumulatively, these *in vivo* results validate the single nucleotide change strategy as a method to reduce toxicity of toxic miRNA triggers.

## DISCUSSION

Testing drugs for human use in two species, generally a rodent and a larger mammal, is a standard procedure for regulatory approval to move forward to clinical studies. Therefore, we set out to test the safety of an RNAi trigger (miHDS1) in mice, which was designed for safety in humans and was shown to be safe in non-human primates (17). While we found no notable toxicity in NHPs when HDS1 was tested in rodents, acute toxicity was noted.

Two different mechanisms can be anticipated by which miHDS1 could induce toxicity in the mouse brain: first, by co-opting and saturating the endogenous miRNA pathway interfering with miRNA regulation, and second, by silencing unintended genes through an miRNA-like mechanism (37,38). Early studies demonstrated *in vivo* RNAi-mediated toxicity due to saturation of the Exportin 5 pathway and buildup of shRNA sequences when expressed in the context of shRNA shuttles (37,38). The use of chemically synthesized double-stranded (siRNA, Dicer-ready siRNAs) or single-stranded RNA nucleic acids (ssRNAs) complexed with specific carriers may circumvent shRNA-associated toxicity, but it will require repeat administration particularly for chronic diseases (33,39–41). Importantly, *in vivo* tolerability can also be attained with the same RNAi trigger when the inhibitory sequences are embedded in an artificial miRNA scaffold (38). This lowers the steady-state levels of the subsequent siRNAs released after Drosha and Dicer cleavage (42). The fact that in this work the noted adverse effects were restricted to miHDS1 and miHDS1v5U, yet their expression was not significantly different from the non-toxic miRNAs suggests that miHDS1 and miHDS1v5U toxicity was not due to high expression and potential RISC pathway saturation. Indeed, higher miRNA expression levels were noted in AAV.miCtl-injected mice, which had phenotypes indistinguishable from mice injected with FB. Therefore, our study supports the use of artificial miRNA shuttles as a system of choice for expressing RNAi triggers in the brain.

*In silico* algorithms have been generated to predict potential off-targets for any given miRNA. In efforts to narrow the possible culprits causing off-target toxicity, we combined the predictive power of the TargetScan and PITA algorithms. This approach identified *Bcl2* among the top off-targeted mRNAs. Bcl2 is a well-known antiapoptotic protein and reduction of *Bcl2* expression by miHDS1 is likely to be associated with adverse effects. Indeed, previous studies have shown that neuronal cell vulnerability and apoptosis can be promoted when expression of specific endogenous miRNA sequences (miR-34a and miR-210) targeting bcl2 mRNA is increased (43,44). Moreover, bcl2 knockdown or knockout in mice induces cell apoptosis and causes degeneration (45).

We found that we could revert the toxicity of the sequence tested, miHDS1, by introducing point mutations into the seed, thereby altering off-target profiles while still maintaining silencing efficacy. This is because single nucleotide mismatches on the seed region can be compensated if extensive complementarity is present beyond the seed, even though some nucleotide positions in the seed regions are more permissive than others (34). Accordingly, not all miHDS1 variants containing a single nucleotide mismatch were equally efficacious against HTT. Changes to the thermodynamic stability of the 5′ end of the active strand may influence miRNA strand loading into the RISC. Also, differences in purine/pyrimidine nucleotide mismatches and the relative mismatch position within the seed region may independently or combinatorially impact silencing efficacy with respect to the original miHDS1 (24,34).

We evaluated whether miHDS1v5U and miHDS1v6A, two of the sequences most effective at silencing HTT, resolved miHDS1 toxicity *in vivo*. Only miHDS1v6A did so. Why is miHDS1v5U still toxic? miHDS1v5U generates a pyrimidine:pyrimidine (U:U) mismatch on miHDS1 off-targeted genes, whereas miHDS1v6A generates a purine:purine (A:G) mismatch. Previous reports have shown that U:U mismatches are more tolerated than A:G mismatches; subsequently, miHDS1v5U will retain the capacity to partially silence miHDS1 off-targets. Accordingly, *Bcl2* mRNA levels remained significantly reduced by miHDS1v5U, but were not by miHDS1v6A.

As an alternative to altering the HDS1 seed, we noted earlier that our control sequence, designed for low off-targeting potential and tolerable in the mouse striata, could be re-engineered to target human HTT mRNA. Following the same strategy for miHDS1, we designed miRNA sequences containing the seed sequence of our control miRNA but re-engineered with a single mismatch in the seed. Among them, miHDss3 significantly silenced human HTT expression confirming that our strategy could be applied to other RNAi triggers. Most importantly, both sequences, when tested in mice, were well tolerated and did not induce neurological deficits, as was noted earlier for the parent HDS1.

These findings highlight and contrast traditional drug development and the newly emerging field of nucleic acid-based medicines. While the goal of all human drug development is safety and efficacy in the target population, in the case of nucleic acid-based medicines, the intended drug interacts directly with the genome and/or the transcripts expressed. Thus, drugs that rely on sequence specificity and optimized for safety in humans will likely interact differently with the genomes of other species, and in particular those of distantly related species such as rodents. On the flip side, if sequences are optimized for safety in rodents, the risk for problems in the context of the human genome may arise.

## SUPPLEMENTARY DATA

Supplementary Data are available at NAR Online.

SUPPLEMENTARY DATA
