# Supplementary material for: Single nucleotide seed modification restores in vivo tolerability of a toxic artificial miRNA sequence in the mouse brain
Source: Nucleic Acids Res. 2014 Oct 20;42(21):13315–27. doi: 10.1093/nar/gku979 (PMC4245975; doi:10.1093/nar/gku979)
Supplement: SUPPLEMENTARY DATA [file supp_42_21_13315__index.html]

Single nucleotide seed modification restores in vivo tolerability of a toxic artificial miRNA sequence in the mouse brain — SUPPLEMENTARY DATA 

# Single nucleotide seed modification restores *in vivo* tolerability of a toxic artificial miRNA sequence in the mouse brain

## SUPPLEMENTARY DATA

**Files in this Data Supplement:**

- SUPPLEMENTARY DATA
- SUPPLEMENTARY DATA
- SUPPLEMENTARY DATA
- SUPPLEMENTARY DATA
- SUPPLEMENTARY DATA
- SUPPLEMENTARY DATA
- SUPPLEMENTARY DATA
