# Supplementary figures and images for: Single nucleotide seed modification restores in vivo tolerability of a toxic artificial miRNA sequence in the mouse brain
Source: Nucleic Acids Res. 2014 Oct 20;42(21):13315–27. doi: 10.1093/nar/gku979 (PMC4245975; doi:10.1093/nar/gku979)

**A**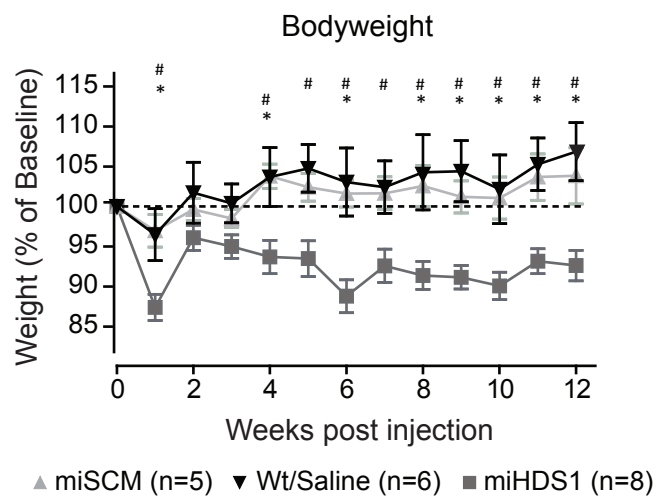**B**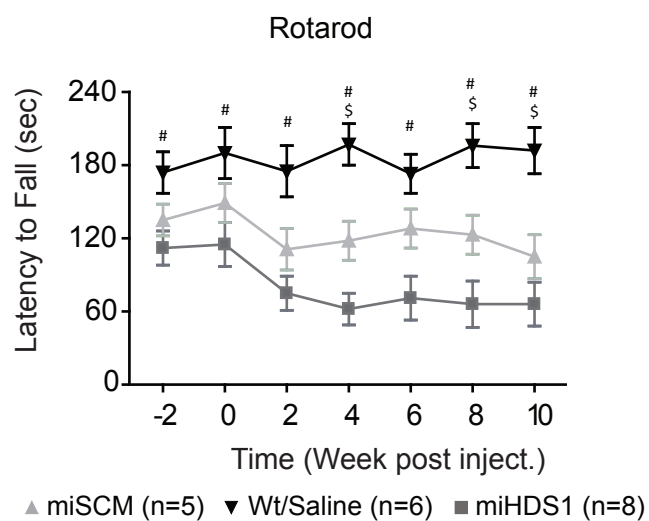

Supplement: SUPPLEMENTARY DATA [file supp_gku979_nar-02020-y-2014-File008.pdf]
