# Supplementary material for: Single nucleotide seed modification restores in vivo tolerability of a toxic artificial miRNA sequence in the mouse brain
Source: Nucleic Acids Res. 2014 Oct 20;42(21):13315–27. doi: 10.1093/nar/gku979 (PMC4245975; doi:10.1093/nar/gku979)
Supplement: SUPPLEMENTARY DATA [file supp_gku979_nar-02020-y-2014-File009.pdf]

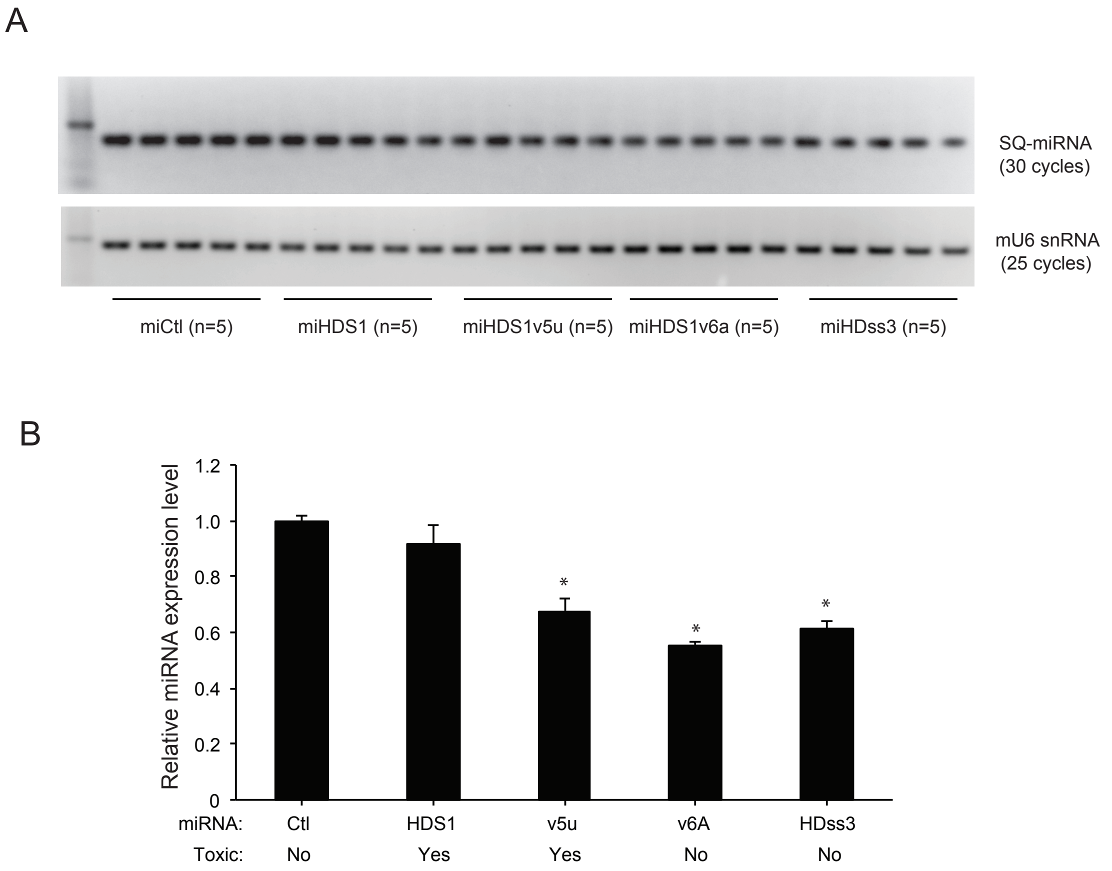

Figure supplemental 2. miRNA expression levels in mouse striatum Total RNA was collected 4 month after injection and miRNA and mU6 snRNA expression was determined by RT-SQ-PCR.. A) Representative photograph of SQ-PCR miRNA products in brain lysates separated on a 3% agarose gel by electrophoresis. B) Semi-quantitative analysis of miRNA levels in mouse striatum. All samples were normalized to *mU6snRNA* and results are the mean ± SEM relative to mice injected with miCtl (n=5 samples; *p<0.01, One way ANOVA followed by a Bonferroni's post-hoc).
